# Formation and function of dauer ascarosides in the nematodes *Caenorhabditis briggsae* and *Caenorhabditis elegans*

**DOI:** 10.1093/g3journal/jkac014

**Published:** 2022-01-30

**Authors:** Sarah M Cohen, Chester J J Wrobel, Sharan J Prakash, Frank C Schroeder, Paul W Sternberg

**Affiliations:** 1 Division of Biology and Biological Engineering, California Institute of Technology, Pasadena, CA 91125, USA; 2 Boyce Thompson Institute and Department of Chemistry and Chemical Biology, Cornell University, Ithaca, NY 14853, USA

**Keywords:** *C. elegans*, *C. briggsae*, ascarosides, dauer, *daf-22*, *glo-1*

## Abstract

The biosynthetic pathways and functions of ascaroside signaling molecules in the nematode *Caenorhabditis elegans* have been studied to better understand complex, integrative developmental decision-making. Although it is known that ascarosides play multiple roles in the development and behavior of nematode species other than *C. elegans*, these parallel pheromone systems have not been well-studied. Here, we show that ascarosides in the nematode *Caenorhabditis briggsae* are biosynthesized in the same manner as *C. elegans* and act to induce the alternative developmental pathway that generates the stress-resistant dauer lifestage. We show that ascr#2 is the primary component of crude dauer pheromone in *C. briggsae*; in contrast, *C. elegans* dauer pheromone relies on a combination of ascr#2, ascr#3, and several other components. We further demonstrate that *Cbr-daf-22*, like its *C. elegans* ortholog *Cel-daf-22*, is necessary to produce short-chain ascarosides. Moreover, *Cbr-daf-22* and *Cel-daf-22* mutants produce an ascaroside-independent metabolite that acts antagonistically to crude dauer pheromone and inhibits dauer formation.

## Introduction

The nematode *Caenorhabditis elegans* has long been used as model organism not only for understanding general biological phenomena but also for the specific questions of nematode development and behavior. However, as we further explore this species, it is important to expand those findings to show they are applicable to other nematodes, beyond *C. elegans*.

The related nematode *Caenorhabditis briggsae* has previously been used to further investigate findings in *C. elegans* (Wang and Chamberlin 2002; Gupta et al. 2007; Hillier et al. 2007). Orthologous protein sequences are about 80% identical between *C. elegans* and *C. briggsae*, similar to the divergence between protein sequences of humans and mouse orthologs (78.5%) (Waterson et al. 2002; Stein et al. 2003). *Caenorhabditisbriggsae* shares many useful traits of *C. elegans* including selfing hermaphroditism, a fully annotated genome, optical transparency, and a similar and short lifecycle (Stein et al. 2003; Gupta et al. 2007). The growth conditions and methods for genetic and behavioral analysis are similar to those for *C. elegans*, and tools developed for *C. elegans* are relatively easy to modify for use in *C. briggsae* if they do not already exist as *C. briggsae* methods (Baird and Chamberlin 2006). In particular, the recent development of an easy and efficient CRISPR method in *C. briggsae* has made further investigation into the genetics and behavior of this species, both for itself and for comparison with *C. elegans*, much more feasible (Cohen and Sternberg 2019; Culp et al. 2020).

One of the most intriguing developmental questions in nematodes is the alternative development of larvae into the dauer diapause stage. This lifestage is thought to be vital to the survival of the species during times of inconsistent or nonexistent food supplies or other limiting conditions, and it is similar to the infective juvenile stage of many parasitic nematodes (Fodor et al. 1983). The dauer lifestage in *C. elegans* and *C. briggsae* is triggered by the confluence of both physical and chemical signals indicating the abundance of food, the concentration of conspecific nematodes in the local area, and the local amount of various dauer-inducing pheromones (Cassada and Russell 1975; Golden and Riddle 1982, 1984). Unlike *C. elegans*, the dauer lifestage in *C. briggsae* is not also triggered by high temperatures (Inoue et al. 2007). Genetic screens for dauer-constitutive and dauer-defective mutants have identified similar genes in both species in the few cases examined, suggesting that the dauer formation pathway is generally conserved between these species (Inoue et al. 2007).

Dauer pheromone was found to comprise various ascarosides, glycosides of the dideoxysugar ascarylose, named for the *Ascaris* parasitic nematodes in which they were first discovered (Jezyk and Fairbairn 1967; Jeong et al. 2005). Ascarosides are synthesized by nematodes throughout their lives and affect not only the developmental dauer decision, but also behaviors including mating, aggregation, and more (Edison 2009; Pungaliya et al. 2009; Srinivasan et al. 2012; Wharam et al. 2017). Nematodes across clades produce different ascaroside profiles indicating that many of these signals are species specific (Choe et al. 2012). A broad range of species responds to nematode-produced ascarosides including fungi (Hsueh et al. 2013) and plants (Manohar et al. 2020).

Ascarosides in *C. elegans* are made modularly, using building blocks derived from cellular waste products including the sugar ascarylose and fatty acid side chains; complexity is increased by modulating the length of the fatty acid-like side chain and attaching head or terminal groups scavenged from neurotransmitters, amino acids, and other readily available materials in the cell (von Reuss et al. 2012). Ascarosides require a shortened fatty acid side chain in order to be functional; this process is done through the peroxisomal beta-oxidation pathway comprising the 4 genes: *Cel-acox-1*, *Cel-maoc-1*, *Cel-dhs-28*, and *Cel-daf-22* (Golden and Riddle 1985; Butcher et al. 2009; von Reuss et al. 2012). Mutants that lack a gene along the beta-oxidation pathway are unable to produce any of the short-chain ascarosides that affect behavior, although they are able to register exogenous ascaroside signals (Butcher et al. 2009). Similar to what is found in other *Caenorhabditis* species, *C. briggsae* has one-to-one orthologs of *Cel-maoc-1* (*Cbr-maoc-1*), *Cel-dhs-28* (*Cbr-dhs-28*), and *Cel-daf-22* (*Cbr-daf-22*) (Harris et al. 2020). *Caenorhabditisbriggsae* also has orthologs of the several *acox-1* genes (e.g. *Cel-acox-1.1* and *Cbr-acox-1.1*) (WormBase WS282; Harris et al. 2020).

In *C. elegans*, further modification to the 4′ position of an ascaroside to form increasingly complex signals occurs in the gut granules (Panda et al. 2017). These are birefringent and autofluorescent lysosome-related organelles in the nematode intestine used to break down and recycle cellular waste (Hermann et al. 2005). Mutants that lack gut granules, such as *Cel-glo-1*, *Cel-glo-3*, or *Cel-apb-3* cannot produce 4′-modified or some terminally modified ascarosides (Rabbitts et al. 2008; Panda et al. 2017; Le et al. 2020). *Cbr-glo-1* is a one-to-one ortholog with its counterpart in *C. elegans* and has been shown to have a similar function; specifically, mutants lack both gut granules and the ability to form 4′-modified ascarosides (Le et al. 2020).

In this study, we show that in *C. briggsae*, *Cbr-daf-22* has a function similar to that of its *C. elegans* ortholog *Cel-daf-22*: both are essential for ascaroside biosynthesis. We combined our investigation of *Cbr-daf-22* with the previously reported mutants of *Cbr-glo-1* to characterize and compare the crude pheromones of AF16, *Cbr-daf-22*, and *Cbr-glo-1*. Through these experiments, we found that the major component of *C. briggsae* dauer-pheromone is ascr#2. We also found antidauer activity in the crude pheromones of both *Cel-daf-22* and *Cbr-daf-22*. Investigation of the *Cel-glo-1* and *Cbr-glo-1* mutant pheromone, which lacks the additional information provided by 4′-modified ascarosides, was found to cause an irregular dauer response curve. Based on these findings, we postulate the existence of an antidauer compound, or class of compounds, that adds further complexity to the intricate and finely tuned system of ascaroside signaling in *Caenorhabditis* species.

## Materials and methods

### *Caenorhabditis briggsae* and *C. elegans* strains and strain maintenance

The Indian strain AF16 was used as a wild type for *C. briggsae* while the Bristol strain N2 was used as a wild type for *C. elegans*. See Table 1 for a list of strains used. All nematode strains were grown on NGM agar plates seeded with *Escherichiacoli* (OP50) at 20°C.

**Table 1.** List of strains.

Genotype	Strain	Source
*C. elegans* wild type	N2	Brenner (1974) and CGC
*Cbr-daf-22*(*sy1524*)	PS8777	This work
*Cbr-daf-22*(*sy1525*)	PS8778	This work
*Cbr-glo-1*(*sy1382*)	PS8515	Le et al. (2020) and Sternberg lab collection
*C. briggsae* wild type	AF16	CGC
*daf-22*(*ok693*)	RB859	The *C. elegans* Deletion Mutant Consortium (2012) and CGC
*glo-1*(*zu391*)	JJ1271	Hermann *et al* (2005) and CGC

CGC, *Caenorhabditis* Genetics Center.

### Construction of *Cbr-daf-22* mutants

Construction of the 2 *Cbr-daf-22* mutants was done using the *C. briggsae* modifications of the universal STOP-IN cassette method as described in Wang et al. (2018) and Cohen and Sternberg (2019). The guide used was AATAGTGCATTAGACGATTG; the forward primer was ATGAGCCCAACCAAGCCAAA; and the reverse primer was CGGCTGGGTATGGAAGCTTT. *Cbr-daf-22*(*sy1524*) was a successful insertion of the universal STOP-IN cassette, while *Cbr-daf-22* (*sy1525*), contains a 34 bp insertion from the *Cbr-dpy-10* locus; both shown below with the flanking sequences underlined.

*sy1524*

GTGAATAGTGCATTAGACGAGGGAAGTTTGTCCAGAGCAGAGGTGACTAAGTGATAAGCTAGCTTGTGGACTAAAATATGCCG

*sy1525*

AAAGAGGCTGTGAATAGTGCTAAATCCGATTTGAAGACCTGTGACACACCGGTAGCTAGCTTATCACTTGTGGACTAAAATATGCCG

### Liquid nematode culturing

Culturing began by chunking *C. briggsae* onto 10 cm NGM plates (each seeded with 800 µl of OP50 *E. coli* grown to stationary phase in Luria–Bertani broth) and incubated at 22°C. Once the food was consumed, each plate was then washed with 25 ml of S-complete medium into a 125 ml Erlenmeyer flask, and 1 ml of OP50 *E. coli* was added (*E. coli* cultures were grown to stationary phase in Luria–Bertani broth, pelleted and resuspended at 1 g wet mass per 1 ml M9 buffer), shaking at 220 rpm and 22°C. After 70 h, cultures were centrifuged at 1,000*g* for 1 min. After discarding supernatant, 24 ml H_2_O was added along with 6 ml bleach, 900 µl 10 M NaOH, and the mixture was shaken for 3 min to prepare eggs. Eggs were centrifuged at 1,000*g*, the supernatant was removed, and the egg pellet was washed with 25 ml M9 buffer twice and then suspended in a final volume of 5 ml M9 buffer in a 50 ml centrifuge tube. Eggs were counted and placed on a rocker and allowed to hatch as L1 larvae for 24 h at 22°C. A total of 75,000 L1 larvae were seeded in 25 ml cultures of S-complete with 1 ml of OP50 and incubated at 220 rpm and 22°C in a 125 ml Erlenmeyer flask. After 72 h, nematodes were spun at 1,000*g* for 5 min and spent medium was separated from nematode body pellet. Separated medium and nematode pellet were flash frozen over liquid nitrogen and then lyophilized. Two biological replicates were grown for each strain. Mutants were grown with parallel wild-type controls, and biological replicates were started on different days.

### Dauer pheromone collection

Crude pheromones of AF16, *Cbr-daf-22(sy1524)*, *Cbr-glo-1(sy1382)*, N2, *Cel-daf-22(ok693)*, and *Cel-glo-1(zu391)* were made using previously described methods (Golden and Riddle 1984; Schroeder and Flatt 2014). Briefly, 1 L of liquid nematode culture (4 flasks of each 250 ml S-complete and 8 ml of OP50 *E. coli*, grown in the same way as in the above liquid cultures) was grown until exhausted. The liquid culture supernatant was separated from the pellet, filtered, and then dried completely. The dried material was extracted using ethanol; the extract was then dried and redissolved in 1 ml of sterilized water.

### Dauer assays

Dauer assays were run using the method described in Lee et al. (2017). The dauer plates were prepared by adding the desired amount of crude pheromone or ascaroside to a 35 × 10 mm plate before adding 2 ml of 2.5% agarose (NG agarose minus Bacto-Peptone). These plates were left to dry for 1 day. Prior to adding any nematodes, the plates were seeded with 10 µl of heat-killed OP50 (OP50 *E. coli* grown in Luria–Bertani broth, spun down, and heated at 97°C for 5 min) in the center of the plate. Ten adult nematodes were placed on the plate and allowed to lay eggs for 3 h. The adult nematodes were removed, and the plates were seeded with an additional 10 µl of OP50 on top of the eggs. The plates were stored in an incubator at 25.5°C for 24 h, after which the plates were quickly checked to confirm normal hatching and L1 development. After a further 24 h in the 25.5°C in the incubator (48 h after the start of the experiment) the total number of nematodes and dauer-stage nematodes were counted for each plate. To continue to ensure that the pheromones did not affect development, plates were kept for a further 24 h after the end of the experiment to check for expected continued development (i.e. that L3s became normal, egg-laying adults and that dauer animals remained in dauer).

### Metabolite extraction

Lyophilized pellet and media samples were crushed and homogenized by shaking with 2.5 mm steel balls at 1,300 rpm for 3 min in 30 s pulses while chilled with liquid nitrogen (SPEX sample prep miniG 1600). Powdered media and pellet samples were extracted with 10 ml methanol in 50 ml centrifuge tubes, rocking overnight at 22°C. Extractions were pelleted at 5,000*g* for 10 min at 4°C, and supernatants were transferred to 20 ml glass scintillation vials. Samples were then dried in a SpeedVac (Thermo Fisher Scientific) vacuum concentrator. Dried materials were resuspended in 1 ml methanol and vortexed for 1 min. Samples were pelleted at 10,000*g* for 5 min and 22°C, and supernatants were transferred to 2 ml HPLC vials and dried in a SpeedVac vacuum concentrator. Samples were resuspended in 100 μl of methanol, transferred into 1.7-ml Eppendorf tubes, and centrifuged at 18,000*g* for 20 min at 4°C. Clarified extracts were transferred to HPLC vials and stored at −20°C until analysis was performed.

### Mass spectrometric analysis

High-resolution LC–MS analysis was performed on a Thermo Fisher Scientific Vanquish Horizon UHPLC System coupled with a Thermo Q Exactive hybrid quadrupole-orbitrap high-resolution mass spectrometer equipped with a HESI ion source. One microliter of extract was injected and separated using a water-acetonitrile gradient on a Thermo Scientific Hypersil GOLD C18 column (150 mm × 2.1 mm 1.9 µm particle size 175 Å pore size, Thermo Scientific) and maintained at 40°C. Solvents were all purchased from Fisher Scientific as HPLC grade. Solvent A: 0.1% formic acid in water; solvent B: 0.1% formic acid in acetonitrile. A/B gradient started at 1% B for 3 min, then from 1% to 100% B over 20 min, 100% for 5 min, then down to 1% B for 3 min. Mass spectrometer parameters: 3.5 kV spray voltage, 380°C capillary temperature, 300°C probe heater temperature, 60 sheath flow rate, 20 auxiliary flow rate, 2.0 spare gas; S-lens RF level 50.0, resolution 240,000, *m*/*z* range 150–1,000 *m*/*z*, AGC target 3e6. The instrument was calibrated with positive and negative ion calibration solutions (Thermo-Fisher) Pierce LTQ Velos ESI pos/neg calibration solutions. Peak areas were determined using Xcalibur 2.3 QualBrowser version 2.3.26 (Thermo Scientific) using a 5-ppm window around the *m*/*z* of interest.

## Results

### Strain construction and phenotype of *Cbr-daf-22*

**Fig. 1.**
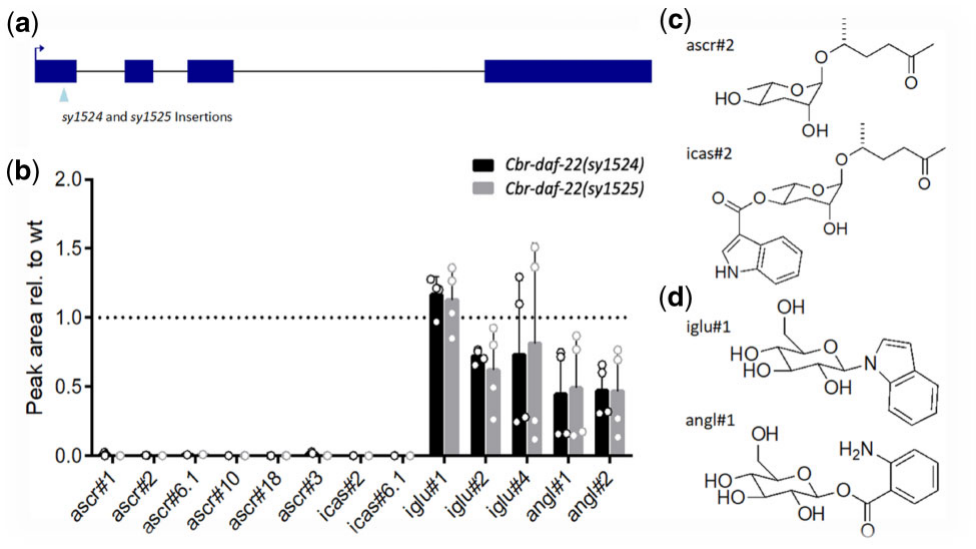
*Caenorhabditis briggsae daf-22* mutant and phenotypes. a) Two *Cbr-daf-22* strains made using the CRISPR/Cas9 triple stop knock-in method to insert the full 43 base pair triple-stop insert (*sy1524*) and a 34 base pair insert (*sy1525*) in the first exon. b) The metabolomes of both *Cbr-daf-22* strains do not produce any functional short-chain ascarosides (e.g. ascr#2) or their 4′ modifications (e.g. icas#2), shown in (c). The *Cbr-daf-22* strains are, however, still able to produce glucoside sugars modified at the 1′ position including indole glucosides (e.g. iglu#1) and anthranilic acid glucosides (e.g. angl#1) as shown in (d).

In previous studies, it has been shown that a *Cbr-glo-1* loss-of-function mutant shares at least part of its phenotype with an orthologous *Cel-glo-1* mutant: neither mutant is able to form gut granules (Hermann et al. 2005; Le et al. 2020). Concomitantly, a *Cbr-glo-1* mutant is also unable to produce complex ascarosides that have been modified with a head group at the 4′ carbon positions (Le et al. 2020). To further explore the similarities between *C. elegans* and *C. briggsae* ascaroside formation, we made 2 mutant strains of *Cbr-daf-22*, ortholog of *Cel-daf-22*, which controls the last step in the *C. elegans* peroxisomal beta-oxidation pathway (Fig. 1a). These *C. briggsae* mutants are unable to form simple (e.g. ascr#2) and modular (e.g. icas#2) short-chain ascarosides (Fig. 1b), the phenotype associated with *Cel-daf-22* mutant metabolomes in *C. elegans* (von Reuss et al. 2012). However, both *Cbr-daf-22* and its ortholog *Cel-daf-22* produce many other types of small molecules, including the previously identified small molecule classes of indole glucosides (iglu) and anthranilic acid glucosides (angl) (Fig. 1, b–d) (Coburn and Gems 2013; Stupp et al. 2013; Le et al. 2020).

### *Caenorhabditis briggsae* crude pheromone dauer assays

**Fig. 2.**
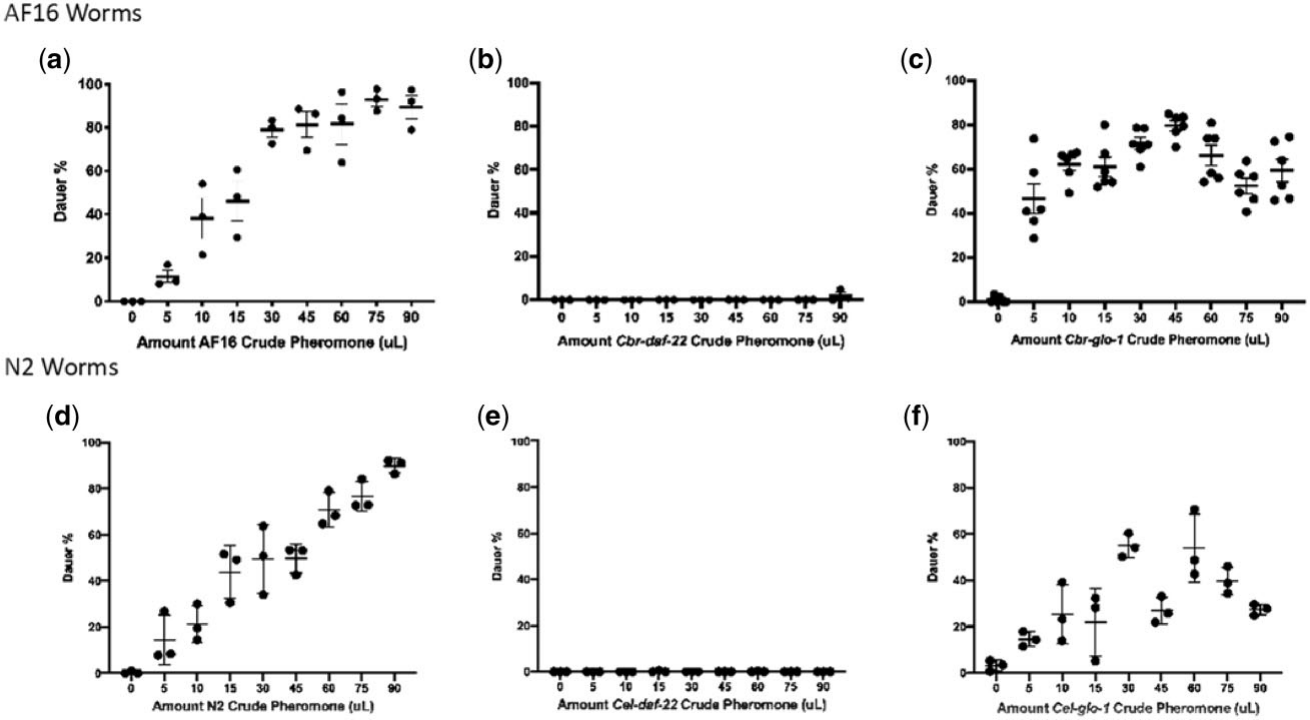
Response to doses of crude pheromone in wild-type *C. briggsae* and *C. elegans*. a) The dauer curve of wild-type nematodes treated with crude pheromone prepared from wild-type nematodes creates a linear increase in dauer percentage with the increase in the amount of pheromone, approaching 100%. b) *Cbr-daf-22*(*sy1524*) crude pheromone produces no dauer-inducing effect on wild-type *C. briggsae* nematodes. c) *Cbr-glo-1*(*sy1382*) crude pheromone produces a dauer curve that remains stable despite changing amounts of dauer pheromone and never approaches 100%. d) When carried out in *C. elegans*, wild-type pheromone, e) *Cel-daf-22* pheromone, and f) *Cel-glo-1* pheromone produce very similar curves to their *C. briggsae* counterparts. Error bars indicate standard deviation.

We performed a series of dauer assays using wild-type (AF16) nematodes to determine how the various innate crude pheromone preparations from *Cbr-glo-1* and *Cbr-daf-22* mutants would affect wild-type nematode dauer formation across a range of concentrations. Wild-type (AF16) crude pheromone exhibited an expected dose–response curve that showed a stable increase in the number of dauers as the amounts of pheromone increased (Fig. 2a). At high pheromone concentrations, almost all nematodes went into dauer. This assay was repeated using crude dauer pheromone from *Cbr-daf-22* (Fig. 2b) and *Cbr-glo-1* mutants (Fig. 2c). As hypothesized, due to its lack of innate short-chain ascarosides, *Cbr-daf-22* crude dauer pheromone was unable to induce dauer formation at any concentration. *Cbr-glo-1* crude pheromone appeared to induce dauer formation at fairly consistent but intermediate levels across a broad range of dosages. A Kolmogorov–Smirnov test showed that the dauer curve of *Cbr-glo-1* pheromone is significantly different from that of AF16 (Supplementary Fig. 1a). Even the highest concentration tested was unable to replicate the dauer-inducing effectiveness of pheromone produced by the wild type. These data suggest that the dauer pheromone in *C. briggsae* are simple (i.e. unmodified) short-chain ascarosides.

These dauer assay experiments were also performed in *C. elegans*, testing the effects on wild-type nematodes of crude pheromone prepared from N2 wild type (Fig. 2d), *Cel-daf-22* (Fig. 2e), and *Cel-glo-1* (Fig. 2f). The same pattern occurs in these assays whereby the N2 pheromone has a typical dose–response curve, the *Cel-daf-22* pheromone induces no dauers, and the *Cel-glo-1* pheromone shows an inconsistent dauer induction effect relative to the amount added. Again, a Kolmogorov–Smirnov test showed that the dauer curve of *Cel-glo-1* pheromone is significantly different from that of N2 (Supplementary Fig. 1b).

### Isolation of main dauer pheromone component in *C. briggsae*

The response of *Cbr-glo-1* mutants to doses of crude pheromone (Fig. 2c) indicates that at least 1 major component of the *C. briggsae* dauer-inducing pheromone must be a simple ascaroside since *Cbr-glo-1* pheromone is able to induce dauer formation. As the *C. briggsae* metabolome primarily consists of ascr#2 and ascr#6.1 and their derivatives (Dong et al. 2016; von Reuss 2018), we were able to delineate the likely major dauer pheromone component to those 2 simple ascarosides. *Caenorhabditisbriggsae* has been shown to respond to *C. elegans* dauer pheromone (Fodor et al. 1983). *C. elegans* dauer pheromone contains ascr#2 (Butcher et al. 2007), making ascr#2 a leading candidate for the dominant dauer pheromone signal in *C. briggsae*.

**Fig. 3.**
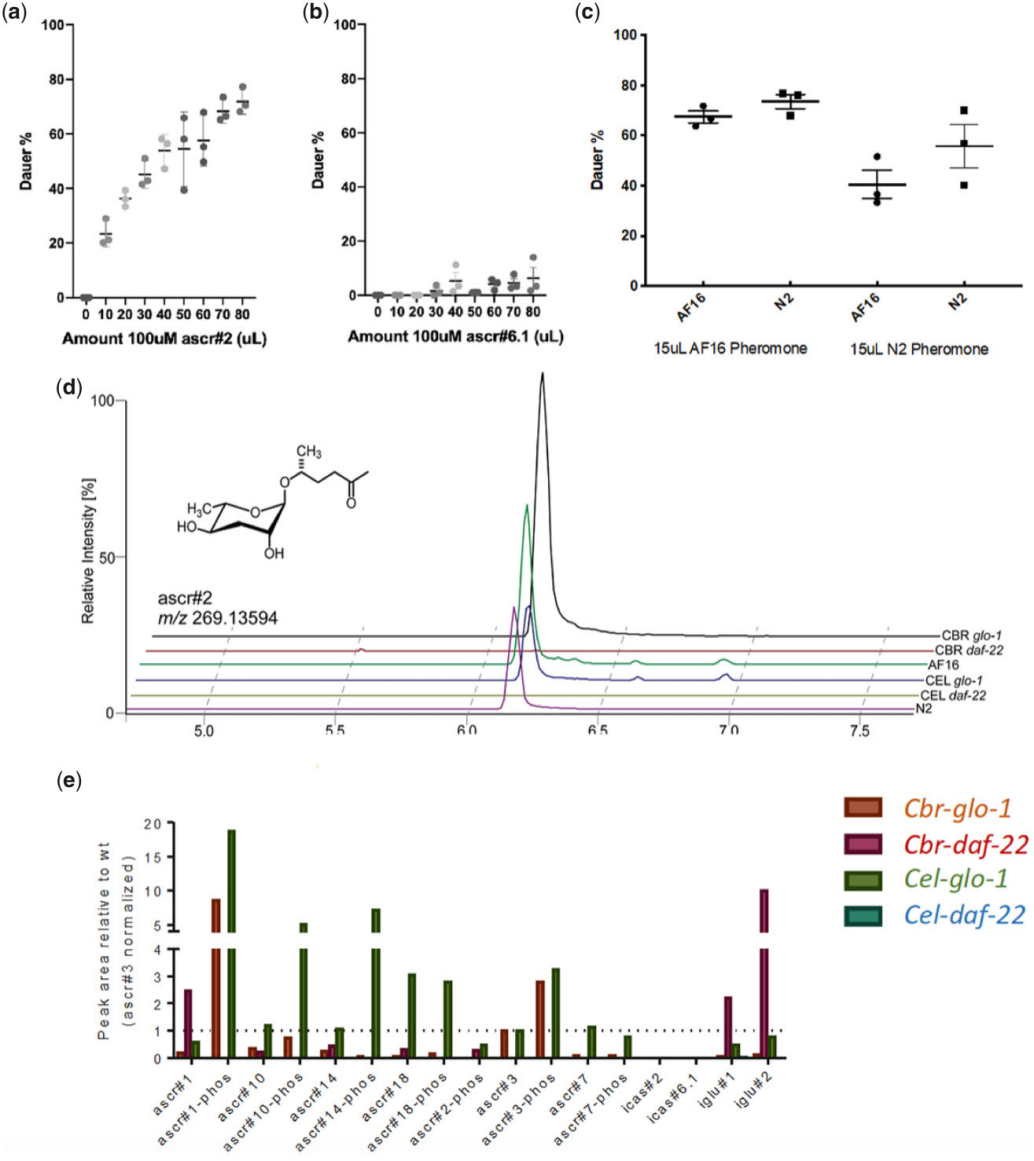
Ascarosides induce dauer formation. a) ascr#2, a main component of the *C. briggsae* metabolome, induces dauer formation increasingly as the number of ascaroside increases, and is a main component of dauer pheromone in *C. briggsae*. b) ascr#6.1, another major component of the *C. briggsae* metabolome, does not significantly induce dauer, even at high concentrations. c) ascr#2 is found in the crude pheromone of *C. briggsae* wild type, *C. elegans* wild type, *Cbr-glo-1*, and *Cel-glo-1*, all of which are able to induce dauer formation; ascr#2 is not found in the crude pheromone of *Cbr-daf-22* or *Cel-daf-22*. d) Crude pheromone affects *C. elegans* and *C. briggsae* similarly except that wild-type *C. briggsae* pheromone appears to be slightly more potent than wild-type *C. elegans* pheromone; *P<* 0.05. e) Crude pheromone from *Cbr-daf-22(sy1524)* shows an increase in the production of modular glucosides compared to wild type, whereas *Cbr-glo-1(sy1382)* crude pheromone shows an increase in the production of phosphorylated ascarosides. Error bars indicate standard deviation.

The dauer pheromone curve for ascr#2 demonstrated that it was the main dauer ascaroside for *C. briggsae* (Fig. 3a). *Caenorhabditisbriggsae* dauer ascarosides were active as low as the micromolar range, consistent with the general amounts of crude pheromone or purified single ascaroside needed to produce dauer formation effects in *C. elegans* (Srinivasan et al. 2012) (Supplementary Fig. 2). In contrast to the dauer-promoting effects of ascr#2, the other main ascaroside produced by *C. briggsae*, ascr#6.1, induced little or no dauer formation, even at high concentrations (Fig. 3b). In agreement with the metabolomics performed above (Fig. 1b), ascr#2 is present in all dauer-inducing crude pheromone tested and is not present in crude pheromone that is unable to induce dauer formation (Fig. 3c).

Comparison of *C. briggsae* and *C. elegans* crude dauer pheromones shows that *C. briggsae* pheromone elicits a slightly higher rate of dauer formation in *C. briggsae* than the same amount of *C. elegans* pheromone (Fig. 3d). However, each pheromone affects the 2 species similarly.

The crude pheromone for *Cbr-daf-22* shows an increase in indole glucosides relative to wild type; this is similar to what is found in the general *Cbr-daf-22* metabolome (Fig. 3e). In contrast, *Cbr-glo-1* and *Cel-glo-1* crude pheromone both show an increase in phosphorylated ascarosides (Fig. 3e).

### *Cbr-daf-22* pheromone has anti-dauer activity

**Fig. 4.**
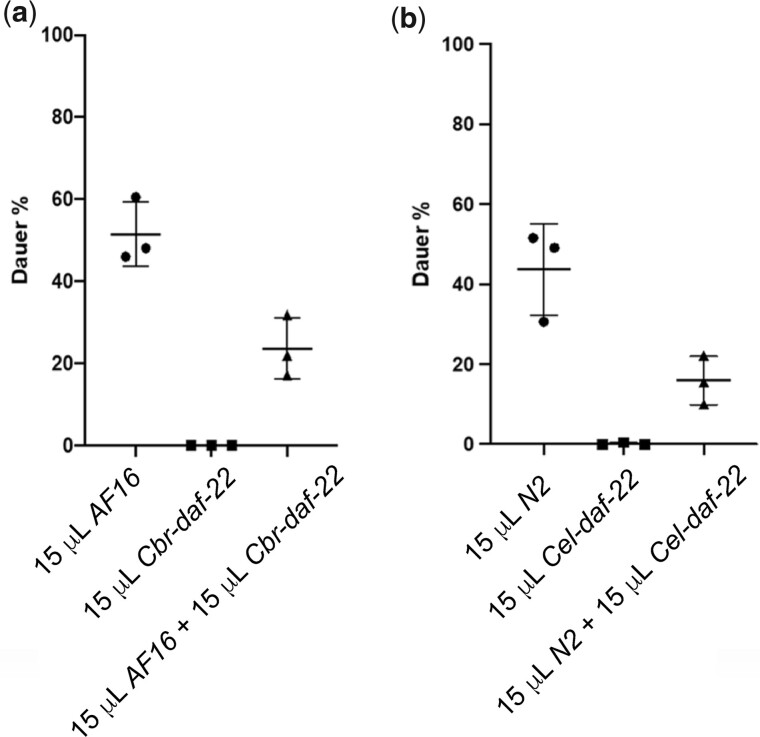
Combinations of crude pheromone are not additive. a) When combined with wild-type *C. briggsae* crude pheromone, *Cbr-daf-22* mutant crude pheromone actively suppresses dauer formation in *C. briggsae* wild-type hermaphrodites. b) The same suppression occurs when wild-type *C. elegans* crude pheromone is combined with *Cel-daf-22* mutant crude pheromone to treat *C. elegans* wild-type hermaphrodites. Error bars indicate standard deviation; *P>* 0.05.

As shown in Fig. 3b, we found that ascr#6.1 had essentially no effect on dauer formation in *C. briggsa*e. However, at high concentrations, ascr#6.1 does induce a small fraction of animals to become dauer larvae. When this was compared to the overall scarcity of dauer formation in response to *Cbr-daf-22* mutant dauer pheromone at all concentrations the *Cbr-daf-22* mutant pheromone appeared to indicate an actively antidauer effect. To test for such an antidauer effect, we combined the crude pheromones of *C. briggsae* wild type and *Cbr-daf-22* to see if such a combination was additive or antagonistic. We found dauer formation was significantly depressed whenever *Cbr-daf-22* pheromone was present (Fig. 4a). When this experiment was performed in *C. elegans* using pheromone produced from the corresponding *C. elegans* strains, the same phenomenon was observed (Fig. 4b). When AF16 and N2 nematodes encountered each other’s *daf-22* pheromone, the same results—no dauer formation—were seen (Supplementary Fig. 3).

## Discussion

### Comparison of ascaroside formation pathways in *C. briggsae* and *C. elegans*

As *C. briggsae* and *C. elegans* are closely related evolutionarily, so too are the basic pathways with which they create their myriad of ascarosides, many which are shown to be nematode-specific communication molecules (reviewed by von Reuss 2018). The *C. briggsae* one-to-one orthologs of *Cel-daf-22* (*Cbr-daf-22*) and *Cel-glo-1* (*Cbr-glo-1*) have been shown to be physiologically and metabolomically equivalent (this work; Le et al. 2020).

*Cel-daf-22* and *Cbr-daf-22* mutants cannot create the biologically relevant short-chain ascarosides used by both nematode species. However, they are still able to produce indole glucosides (iglu) and anthranilic acid glucosides (angl) at levels comparable to, or slightly lower than wild type. Functionally, the absence of short-chain ascarosides in *Cel-daf-22* and *Cbr-daf-22* crude pheromone causes a complete lack of dauer formation under the same conditions where increased amounts of *C. briggsae* or *C. elegans* wild-type crude pheromone, respectively, causes a linear increase in dauer formation.

*Cel-glo-1* and *Cbr-glo-1* mutants have previously been shown to lack gut granules and the subsequent ability to form 4′-modified ascarosides (Panda et al. 2017; Le et al. 2020). Functionally, this lack appears to also create an imbalance in the fine-tuned system of dauer-formation communication signaling and causes *Cbr-glo-1* and *Cel-glo-1* mutant crude pheromone to have unusual dose response curves where the amount of dauer formation appears to be independent of the amount of pheromone encountered. As phosphorylated ascarosides are upregulated in both *Cel-glo-1* and *Cbr-glo-1* (Fig. 3e), they are possible candidates for this effect.

With these similarities of the main ascaroside-forming pathways, we can infer that other major components of the ascaroside formation pathways, such as the remainder of the beta-oxidation pathway, are likely to be similarly conserved. We also expect to see further conservation in the major components of ascaroside formation between *C. elegans* and other nematode species, especially when their ascaroside profiles overlap.

### Isolation of primary dauer pheromone component in *C. briggsae*

*Caenorhabditis* *briggsae* releases fewer types of known ascarosides than *C. elegans* and, subsequently, has seemingly fewer behaviors regulated by these ascarosides (Dong et al. 2016). The pathways that are conserved between the 2 species indicate their importance to those behaviors.

We confirmed that ascr#2 is the main component of dauer pheromone in *C. briggsae*. Thus, ascr#2 acts functionally the same in both *C. briggsae* and *C. elegans*. Combined with the conserved ascaroside formation pathways, this finding indicates that it may be an ascaroside originally used by the evolutionary ancestor of both species. However, the 2 species then diverge in other dauer-inducing ascaroside signals such as ascr#3, which is another major component of *C. elegans* dauer pheromone (Butcher et al. 2007), but which is absent in the *C. briggsae* metabolome.

*Caenorhabditis* *elegans* and *C. briggsae* not only are closely related in the *elegans* group of the *Caenorhabditis* genus, but also have overlapping habitats (Cutter et al. 2006). The 2 species have diverged climatically and temporally with *C. briggsae* preferring warmer climates and seasons than *C. elegans* (Félix and Duveau 2012). But this divergence likely did not change their underlying communication needs when it comes to basic dauer-ascaroside messaging. Overlying yet distinct ascaroside profiles may also indicate that, in the wild, their pheromone signaling may be mutually beneficial to both species using broad-strokes signals such as ascr#2. However, we know that indole-modified ascaroside biosynthesis and subsequent behavior is highly species dependent (Dong et al. 2016). Further refinement using species-specific signaling would allow the nematodes to give the best adaptive advantages to their own brethren. These different signals also would allow the nematodes to ignore signals that indicate imperfect conditions for other species when conditions for their own species (temperature proclivities, for example) are ideal.

It has been reported that an indole-modified version ascr#2, icas#2, acts as a sex pheromone in *C. briggsae* (Dong et al. 2016). The unmodified pheromone may be used as an indication of negative reproductive conditions and thus a dauer-promoting signal while the further modification indicates positive reproductive conditions.

### Antidauer effects of *Cbr-daf-22* and *Cel-daf-22* dauer pheromone

Although the absence of short-chain ascarosides and their dauer-inducing signals staves off dauer-formation under favorable developmental conditions, the almost complete lack of dauer formation in *Cbr*-*daf-22* and *Cel-daf-22* samples indicated something more than a simple absence of dauer ascarosides. Subsequently, we found that *Cbr-daf-22* and *Cel-daf-22* pheromone actively depresses dauer formation, even when in the presence of dauer-promoting crude pheromone from wild-type nematodes.

Our findings indicate that *Cbr-daf-22* and *Cel-daf-22* metabolomes contain either a specific antidauer compound or a compound that acts as a receptor antagonist. Potential candidates for either process include the angl or iglu families of glycosides, as these small molecules are highly abundant in *Cbr-daf-22* and *Cel-daf-22* metabolomes. The iglu family of glycosides is especially interesting as a potential antidauer signal as previous studies have shown these small molecules represent detoxification products of indole derived from *E. coli*, the primary food source of lab-cultured nematodes (Stupp et al. 2013). It would also help explain the idea that if there is some food, there will never be 100% dauer even at extremely high concentrations of dauer pheromone (Golden and Riddle 1984). As the *Cel-daf-22* and *Cbr-daf-22* pheromones both are able to suppress dauer in both *C. briggsae* and *C. elegans*, the antidauer pheromone for both species is hypothesized to contain at least 1 identical compound.

In addition to food, population density, and known dauer pheromones, it is clear that the dauer-decision system is further adjusted through the use of additional pheromone signaling. Thus, the dauer decision is not simply made under unfavorable conditions opposite to the standard L3 reproductive stage—instead there is an active antidauer signal that adds to the complexity of the finely tuned dauer-decision process in *C. elegans* and *C. briggsae.*

## Data availability

Strains are available upon request. The authors affirm that all data necessary for confirming the conclusions of the article are present within the article, figures, and tables.

Supplemental material is available at *G3* online.

## Supplementary Material

jkac014_Supplementary_Data
