# Supplementary material for: Formation and function of dauer ascarosides in the nematodes Caenorhabditis briggsae and Caenorhabditis elegans
Source: G3 (Bethesda). 2022 Jan 30;12(3):jkac014. doi: 10.1093/g3journal/jkac014 (PMC8895998; doi:10.1093/g3journal/jkac014)
Supplement: jkac014_Supplementary_Data [file jkac014_supplementary_data.pdf]

Supplemental Information

SI Figure 1: Kolmogorov-Smirnov tests comparing wildtype to *glo-1* dauer curves in *C. elegans* and *C. briggsae*.

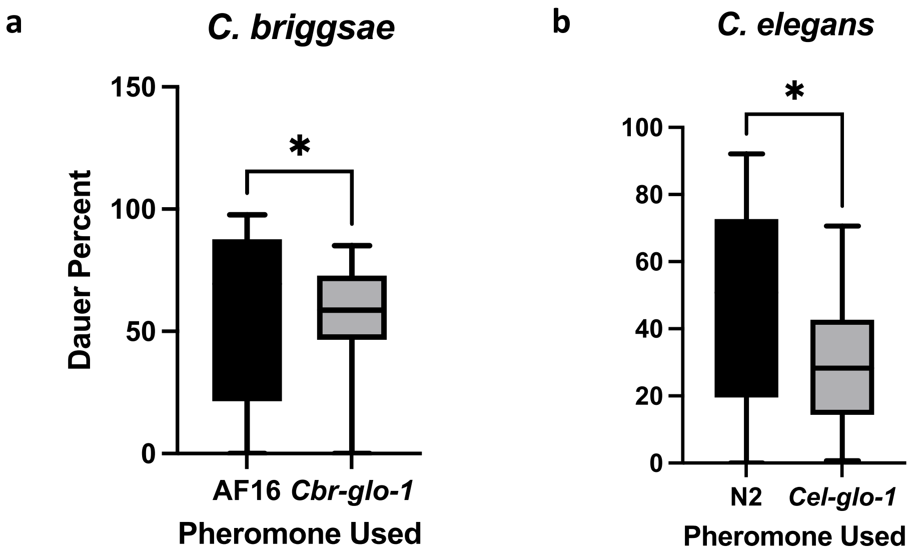

SI Fig. 1 Kolmogorov-Smirnov (KS) nonparametric tests show that the wildtype dauer curves shown in Figure 2 are significantly different from their respective *glo-1* dauer curves. a) KS comparison of *C. briggsae* dauer curves between AF16 pheromone and *Cbr-glo-1* pheromone is significantly different at $P<0.05$. b) Similarly, KS comparison of *C. elegans* dauer curves between N2 pheromone and *Cel-glo-1* pheromone is significantly different at $P<0.05$.

SI Figure 2: Active concentrations of ascr#2 in *C. briggsae*

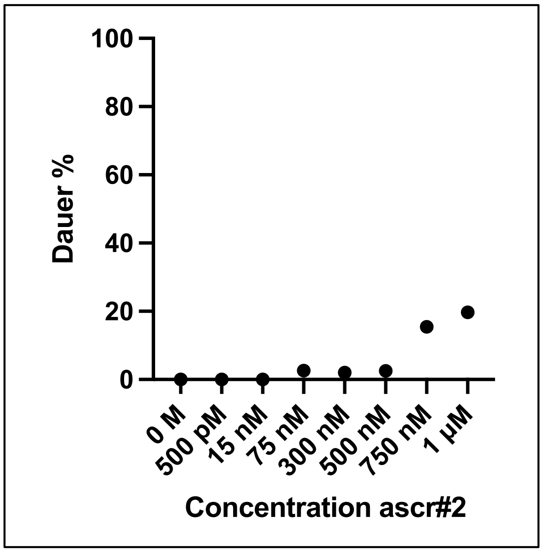

SI Fig. 2. Ascr#2 begins to induce dauer development in *C. briggsae* between 500 nM and 750 nM. Dauer assays were performed as described in Materials and Methods.

SI Figure 3: Effects of *Cbr-daf-22* and *daf-22* pheromone on AF16 and N2 nematodes.

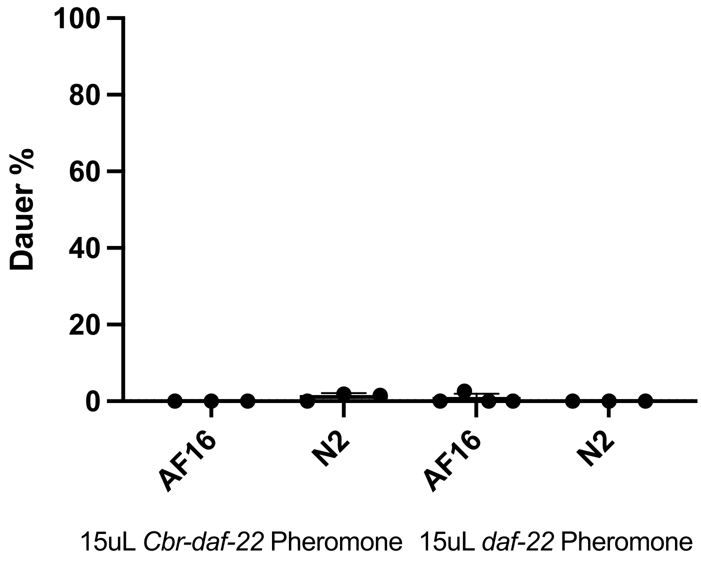

SI Fig. 3. *Cbr-daf-22* and *daf-22* pheromone affects *C. elegans* and *C. briggsae* similarly. Dauer assays were performed as described in Materials and Methods. Error bars indicate standard deviation.
